# Comparison of clinical outcomes between laparoscopic and open surgery for left-sided colon cancer: a nationwide population-based study

**DOI:** 10.1038/s41598-019-57059-6

**Published:** 2020-01-09

**Authors:** Yu-Min Huang, Yuan-Wen Lee, Yan-Jiun Huang, Po-Li Wei

**Affiliations:** 1 Department of Surgery, School of Medicine, College of Medicine, Taipei Medical University, Taipei, Taiwan; 2 Division of Gastrointestinal Surgery, Department of Surgery, Taipei Medical University Hospital, Taipei Medical University, Taipei, Taiwan; 3 Cancer Research Center, Taipei Medical University Hospital, Taipei Medical University, Taipei, Taiwan; 4 Department of Anesthesiology, Taipei Medical University Hospital, Taipei, Taiwan; 5 Department of Anesthesiology, School of Medicine, College of Medicine, Taipei Medical University, Taipei, Taiwan; 6 Division of Colorectal Surgery, Department of Surgery, Taipei Medical University Hospital, Taipei Medical University, Taipei, Taiwan; 7 Translational Laboratory, Department of Medical Research, Taipei Medical University Hospital, Taipei Medical University, Taipei, Taiwan; 8 Graduate Institute of Cancer Biology and Drug Discovery, Taipei Medical University, Taipei, Taiwan

**Keywords:** Cancer therapy, Outcomes research

## Abstract

The role of laparoscopic surgery for left-sided colon cancer has been supported by the results of randomized controlled trials. However, its benefits and disadvantages in the real world setting should be further assessed with population-based studies.The hospitalization data of patients undergoing open or laparoscopic surgery for left-sided colon cancer were sourced from the Taiwan National Health Insurance Research Database. Patient and hospital characteristics and perioperative outcomes including length of hospital stay, operation time, opioid use, blood transfusion, intensive care unit (ICU) admission, and use of mechanical ventilation were compared. The overall survival was also assessed. Patients undergoing laparoscopic surgery had shorter hospital stay (*p* < 0.0001) and less demand for opioid analgesia (*p* = 0.0005). Further logistic regression revealed that patients undergoing open surgery were 1.70, 2.89, and 3.00 times more likely to have blood transfusion, to be admitted to ICU, and to use mechanical ventilation than patients undergoing laparoscopic surgery. Operations performed in medical centers were also associated with less adverse events. The overall survival was comparable between the 2 groups.With adequate hospital quality and volume, laparoscopic surgery for left-sided colon cancer was associated with improved perioperative outcomes. The long-term survival was not compromised.

## Introduction

Colorectal cancer is a worldwide health problem. It is the 3rd most commonly diagnosed malignancy in the world and is also one of leading causes of cancer death^1^. Laparoscopic colectomy was introduced in 1991^2^. Initially, it was not widely accepted for cancer treatment because of technical difficulties such as working in multiple intra-abdominal quadrants, ligation of vessels and re-establishment of intestinal continuity as well as oncological concerns including retrieval of lymph nodes, surgical resection margin and survival results^3^. These controversies gradually settled with the accumulation of experience and advance in technology. Through medial-to-lateral approach, the difficulty of multi-quadrant working was lessened. New energy devices such as bipolar sealers and ultrasonic shears made ligation of vessels easier. For oncologic concerns, laparoscopic colectomy was accepted as an alternative surgical approach for colon cancer after positive outcomes from several multi-center prospective randomized trials (RCTs)^4–8^. Since then, further evidence has accumulated to support the feasibility, safety, and benefits of the laparoscopic surgery for colorectal cancer^9–11^.

Although RCTs provide high level of evidence, there were some problems with surgical RCTs such as learning curves of new techniques, difficulty of surgical quality monitoring, blinding, and comprehensiveness of follow-up. The results obtained in the strictly controlled settings where the RCTs were performed could not always be applied readily to the real world condition^12^. In fact, the conclusions made from RCTs should be assessed further in population-based studies^13^. Moreover, most of the patients in the mentioned RCTs were from the Western regions^5–8^. Whether the conclusion made could be applied to Asian population needs further confirmation.

Taiwan National Health Insurance (NHI) was initiated in 1995. It is a single-payer payment system with government as the sole insurer and more than 99% of Taiwan’s 23 million population have been enrolled. With Taiwan’s National Health Insurance Research Database (NHIRD), we previously demonstrated that laparoscopic right hemicolectomy for transverse colon cancer reduced risk of post-operative pulmonary complications which might be attributed to the obviation of a large upper abdominal incision of the conventional procedure^14^.

However, there was some difference between the nature of procedures for right-sided and left-sided colon cancer. For instance, the incision for sigmoid colon cancer surgery was made over lower abdomen and therefore may be associated with lower intensity of post-operative pain. In addition, evidence on the difference in incidence and severity of post-operative pulmonary complications between laparoscopy and laparotomy for left-sided colon cancer is still scanty. Therefore we performed this study to compare the clinical outcomes of laparoscopic and open surgery for left-sided colon cancer through a nationwide database.

## Methods

### Database

The hospitalization data for this study were sourced from the NHIRD. The NHIRD was published by the National Health Research Institute and was derived from the system of the Taiwan NHI. Taiwan NHI has a number of unique characteristics: universal coverage, a single-payer payment system with the government as the sole insurer, comprehensive benefits, access to any medical institution of the patient’s choice, low out-of-pocket payment, and a wide variety of providers well distributed throughout the country. This dataset includes medical claims data such as medical expenditures, patients’ demographics, diagnostic codes, operation codes, et cetera from Taiwan NHI program. This study was exempted from full review by the Taipei Medical University Institutional Review Board since the NHIRD consists of de-identified secondary data released to the public for research purposes. For this same reason, informed consent was not required. The use of data and methods of data processing in this study were in accordance with the relevant guidelines and regulations of the Taiwan National Health Insurance Research Database.

### Study sample

After excluding 46 patients who have been diagnosed with other primary cancer, we identified 667 descending colon cancer and sigmoid colon cancer patients undergoing a left hemicolectomy or a sigmoid colon resection between January 2007 and December 2013 based on the ICD-9-CM procedure code 45.75 and 45.76 and ICD-9 disease code 153.2 and 153.3. Of these 667 cases, we further identified those who underwent laparoscopic or open surgeries by the additional ICD-9-CM procedure code 54.21 and the specific procedure codes of NHIRD. As a result, 521 and 146 patients underwent open and laparoscopic surgery for left-sided colon cancer, respectively, during this period.

### Key variables of interest

All the variables used in this study were retrieved from inpatient claims. The primary study outcomes were “length of hospital stay”, “operation time”, “opioid use”, “blood transfusion”, “intensive care unit (ICU) admission”, and “use of mechanical ventilation”. The independent variable of interest was whether or not a laparoscopy was used for left-sided colon cancer resection. In this study, we also took potential confounders including the characteristics of patients and hospitals into consideration in the regression modeling. Patient characteristics included age, gender, monthly income, residence area, and presence of major comorbidities (congestive heart failure, cerebrovascular disease, chronic pulmonary disease, etc.). Hospitals were divided into medical centers, metropolitan hospitals, and local community hospitals according to their teaching and service statuses.

### Statistical analysis

The SAS System for Windows 9.4 (SAS Institute Inc, Cary, NC, USA) was used to perform the analyses in this study. We performed Pearson χ^2^ tests to examine the differences between patients who underwent a laparoscopic or open left-sided colon cancer resection, in terms of characteristics of patients and hospitals. Logistic regressions were carried out for clinical outcomes, including blood transfusion, ICU admission, and mechanical ventilation. The variables with p value < 0.2 in the univariate analysis were included in the multivariable logistic regression model. The cumulative probability of survival for patients receiving laparoscopic and open surgery was estimated using a Kaplan-Meier estimator. The log-rank test was used to compare the survival curves between different groups. A two-sided p value of less than or equal to 0.05 was considered to be statistically significant.

To ameliorate the possible bias stemming from the retrospective nature of the study, we performed further propensity score matching with a 2: 1 ratio. Propensity scores were determined by a logistic regression model of the covariates including: age, sex, monthly income, residence area, comorbidities, and hospitals. Each patient in the laparoscopic group was individually matched to 2 patients in the open group by using the propensity scores. Analysis of the outcome parameters of these patients was then performed in the same manner as in the unmatched patients.

### Ethical approval

This study was approved by instutional ethical comittee.

## Results

The baseline characteristics such as age, sex, income, residence area, comorbidity, and the statuses of hospitals where the operations were performed were similarly distributed between the patients undergoing an open or laparoscopic left-sided colon cancer resection (Table 1). In contrast, significant difference was observed between the two groups of patients with regard to perioperative outcomes. As shown in Table 2, significantly greater proportion of patients undergoing laparoscopic surgery stayed in the hospital for less than 10 days (28.2% in the open group vs. 51.4% in the laparoscopic group, p < 0.0001). The operation time took longer than 4 hours more commonly in patients undergoing laparoscopic surgery (34.0% in the open group vs. 58.2% in the laparoscopic group, p < 0.0001). Patients in the laparoscopic group received blood transfusion less frequently (29.6% in the open group vs. 19.9% in the laparoscopic group, p = 0.02) and received less opioid perioperatively (51.3% in the open group vs. 34.9% in the laparoscopic group receiving >15 morphine equivalent dose). Patients undergoing laparoscopic procedures also had significantly lower likelihood of ICU admission (27.6% in the open group vs. 13.7% in the laparoscopic group, p = 0.0005) and mechanical ventilation (20.9% in the open group vs. 8.9% in the laparoscopic group, p = 0.0009) than did patients undergoing open procedures.

**Table 1:** Characteristics of patients according to types of surgery.

Characteristics	Total (N = 667)		Open (N = 521)		Laparoscopic (N = 146)		*p* value
	Mean (SD) or N (%)		Mean (SD) or N (%)		Mean (SD) or N (%)		
Age, years	65.1	(12.9)	65.1	(13.2)	65.2	(12.2)	0.99
<55	152	(22.8)	124	(23.8)	28	(19.2)	0.32
55–64	169	(25.3)	124	(23.8)	45	(30.8)	
65–74	160	(24.0)	125	(24.0)	35	(24.0)	
≥75	186	(27.9)	148	(28.4)	38	(26.0)	
Male	388	(58.2)	298	(57.2)	90	(61.6)	0.34
Monthly income (NTD)							0.14
0 (Dependent)	212	(31.8)	168	(32.3)	44	(30.1)	
1–20000	145	(21.7)	106	(20.4)	39	(26.7)	
20000–29999	201	(30.1)	166	(31.9)	35	(24.0)	
≥30000	109	(16.3)	81	(15.6)	28	(19.2)	
Residence area							0.27
Central city	291	(43.6)	224	(43.0)	67	(45.9)	
Suburban	171	(25.6)	141	(27.1)	30	(20.6)	
Countryside	205	(30.7)	156	(29.9)	49	(33.6)	
Comorbidity							
Congestive heart failure	21	(3.2)	18	(3.5)	3	(2.1)	0.59
Cerebrovascular disease	54	(8.1)	38	(7.3)	16	(11.0)	0.15
Chronic pulmonary disease	62	(9.3)	45	(8.6)	17	(11.6)	0.27
Renal disease	25	(3.8)	19	(3.7)	6	(4.1)	0.79
Liver disease	44	(6.6)	34	(6.5)	10	(6.9)	0.89
Metastasis	21	(3.2)	16	(3.1)	5	(3.4)	0.83
Hypertension	284	(42.6)	216	(41.5)	68	(46.6)	0.27
Diabetes mellitus	115	(17.2)	82	(15.7)	33	(22.6)	0.052
Hospital							
Medical center	352	(52.8)	280	(53.7)	72	(49.3)	0.37
Metropolitan hospital	289	(43.3)	219	(42.0)	70	(48.0)	
Local community hospital	26	(3.9)	22	(4.2)	4	(2.7)

**Table 2:** Perioperative outcomes of the patients.

Variables	Total (N = 667)		Open (N = 521)		Laparoscopic (N = 146)		*p* value
	N (%)		N (%)		N (%)		
Length of hospital stay (Days)							<0.0001
≤10	222	(33.3)	147	(28.2)	75	(51.4)	
>10	445	(66.7)	374	(71.8)	71	(48.6)	
Surgery time (Hours)							<0.0001
≤4	405	(60.7)	344	(66.0)	61	(41.8)	
>4	262	(39.3)	177	(34.0)	85	(58.2)	
Opioid use (MEQ)							0.0005
≤15	349	(52.3)	254	(48.8)	95	(65.1)	
>15	318	(47.7)	267	(51.3)	51	(34.9)	
Blood transfusion							0.02
Yes	183	(27.4)	154	(29.6)	29	(19.9)	
No	484	(72.6)	367	(70.4)	117	(80.1)	
ICU admission							0.0005
Yes	164	(24.6)	144	(27.6)	20	(13.7)	
No	503	(75.4)	377	(72.4)	126	(86.3)	
Mechanical ventilation							0.0009
Yes	122	(18.3)	109	(20.9)	13	(8.9)	
No	545	(81.7)	412	(79.1)	133	(91.1)	

Abbreviation: MEQ, morphine equivalent dose.

**Figure 1:**
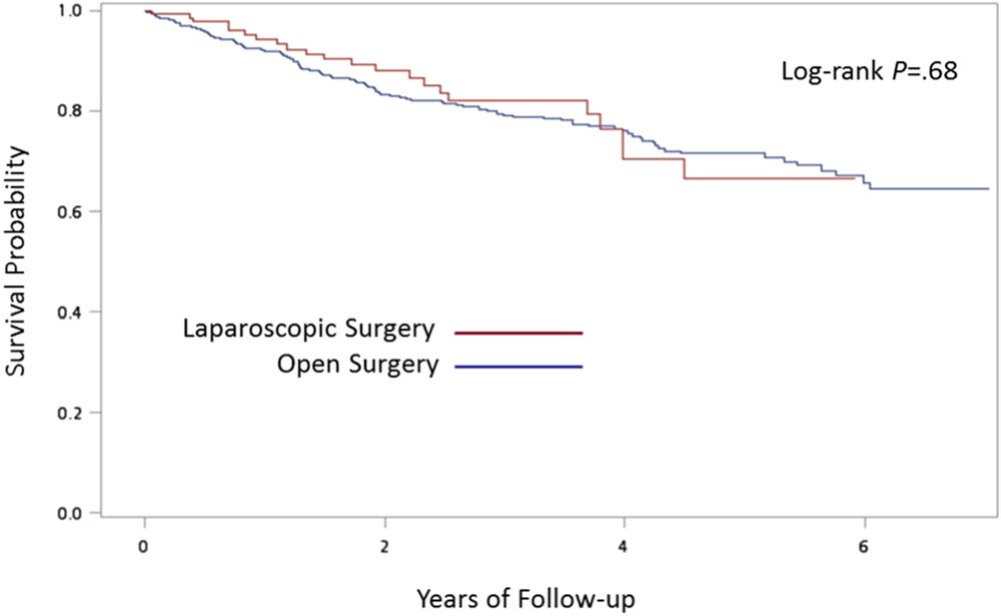
Kaplan-Meier curves of overall survival.

After adjusting for variables listed in Table 3, logistic regression revealed that patients who underwent an open resection were 1.70 (95% CI 1.06–2.75), 2.89 (95% CI 1.67–5.02), and 3.00 (95% CI 1.59–5.67) times more likely to have blood transfusion, to be admitted to ICU, and to use mechanical ventilation than patients undergoing a laparoscopic resection (Table 3). In addition, patients aged from 65 to 74 years were 2.52 (95% CI 1.32–4.82) and 2.46 (95% CI 1.18–5.12) times more likely to have ICU admission and mechanical ventilation than patients younger than 55 years. The adjusted odds ratios were even higher for patients older than 75 years. They were 2.83 (95% CI 1.62–4.93), 5.64 (95% CI 3.00–10.61), and 4.86 (95% CI 2.40–9.81) times more likely to experience blood transfusion, ICU admission, and mechanical ventilation than patients younger than 55 years. Patients with underlying renal disease or metastasis were 2.68 (95% CI 1.10–6.56) and 3.12 (95% CI 1.22–8.03) times more likely to have blood transfusion, respectively. Patients with metastasis were also 3.95 (95% CI 1.46–10.68) times more likely to use mechanical ventilation. Those patients who received operations in metropolitan hospitals were 1.53 (95% CI 1.05–2.23), 2.90 (95% CI 1.93–4.36), and 1.70 (95% CI 1.10–2.61) times more likely to be associated with adverse clinical outcomes including blood transfusion, ICU admission, and mechanical ventilation than patients undergoing operations in medical centers. Those who were operated on in community hospitals were also 4.71 (95% CI 1.95–11.39) times more likely to have blood transfusion. However, the overall survival of the patients seemed not to be different between those receiving open and laparoscopic descending and sigmoid colon cancer resections (Fig. 1).

**Table 3:** Relative risks of clinical outcomes.

Variables	Blood transfusion	ICU admission	Mechanical ventilation
	aOR (95% CI)	aOR (95% CI)	aOR (95% CI)
Open surgery	1.70 (1.06–2.75)	2.89 (1.67–5.02)	3.00 (1.59–5.67)
**Age, years**			
<55	1	1	1
55–64	0.85 (0.48–1.52)	1.00 (0.50–1.99)	1.27 (0.59–2.72)
65–74	1.31 (0.74–2.34)	2.52 (1.32–4.82)	2.46 (1.18–5.12)
≥75	2.83 (1.62–4.93)	5.64 (3.00–10.61)	4.86 (2.40–9.81)
Male	0.48 (0.33–0.70)	1.13 (0.76–1.69)	0.96 (0.63–1.49)
**Comorbidity**			
Congestive heart failure	1.29 (0.46–3.61)	1.66 (0.59–4.67)	2.46 (0.91–6.68)
Cerebrovascular disease	0.88 (0.44–1.74)	1.54 (0.80–2.98)	1.35 (0.67–2.72)
Chronic pulmonary disease	1.29 (0.70–2.36)	0.88 (0.47–1.67)	1.02 (0.52–2.01)
Renal disease	2.68 (1.10–6.56)	1.54 (0.59–4.04)	1.31 (0.48–3.61)
Liver disease	0.90 (0.43–1.91)	0.97 (0.44–2.15)	1.11 (0.49–2.53)
Metastasis	3.12 (1.22–8.03)	1.31 (0.43–4.01)	3.95 (1.46–10.68)
Hypertension	0.87 (0.57–1.31)	0.80 (0.52–1.23)	0.82 (0.51–1.31)
Diabetes mellitus	1.29 (0.78–2.15)	1.41 (0.83–2.40)	1.35 (0.77–2.37)
**Hospital**			
Medical center	1	1	1
Metropolitan hospital	1.53 (1.05–2.23)	2.90 (1.93–4.36)	1.70 (1.10–2.61)
Local community hospital	4.71 (1.95–11.39)	1.63 (0.62–4.28)	0.68 (0.21–2.28)

Abbreviation: aOR, adjusted odds ratio.

The propensity score matching process yielded 276 patients in the open surgery group and 138 patients in the laparoscopic surgery group. Analysis of the outcomes of these groups of patients attained results concurrent with the comparison of the original unmatched groups of patients (see Supplementary Tables S1 and S2).

## Discussion

Through investigation of a nationwide and population-based database, we demonstrated that comparing with patients receiving open surgery for left-sided colon cancer, patients receiving laparoscopic surgery had shorter hospital stay, less demand for opioid analgesia, and lower risk of blood transfusion, ICU admission, and dependence on mechanical ventilation. Aside from these benefits in perioperative outcomes, the overall survival was comparable between patients undergoing laparoscopic and open procedures.

Several RCTs have demonstrated that laparoscopic surgery for colon cancer is a safe and feasible procedure. The quality of the surgical specimen and the long-term oncological outcomes of laparoscopic surgery are equivalent to those of open surgery; however, recovery, physiological function, and other short-term outcome measures are improved with laparoscopic approach^4,7,8,10,11^. However, most participating hospitals in these trials were specialized centers with extensive experience in laparoscopic colon cancer surgery and the studies were performed in strictly controlled environment, limiting the generalizability of their results. In our study, laparoscopic left-sided colon cancer resection was associated with favorable short-term outcomes without compromising the overall survival, indicating that the benefits of laparoscopic surgery observed in clinical trials were preserved not only in the real world but also in the East Asian population.

However, it could also be noted that operations performed outside of medical centers were associated with increased risk of adverse clinical outcomes in our study. It has been reported that hospital volume, surgeon volume, and the rate of laparoscopic surgery may affect the outcome of colorectal surgery. Higher hospital and surgeon volume and higher laparoscopy rates are generally associated with better outcomes after laparoscopic surgery for colorectal cancer^15,16^. In our study, the proportion of laparoscopic left-sided colon cancer resection was similar among hospitals of various statuses, indicating the high penetrance of this procedure. Under such circumstances, surgery performed in medical centers was associated with significantly lower risk of adverse clinical outcomes than surgery performed in metropolitan hospitals. This observation indicated that hospital volume and capability remained a significant determinant of clinical outcomes after colon cancer surgery.

Pulmonary complications including atelectasis, pneumonia, and acute respiratory failure are among the most common causes of morbidity in the post-operative period. The reported incidence ranged from 17% to 88%, depending on the definition, patient population, and surgical procedures^17^. These adverse events lead to prolonged ICU admission, ventilator dependence, and hospital stay^18^. Dependence on mechanical ventilation may further lead to various systemic adverse effects including lower cardiacoutput^19^, increased risk of gastrointestinal complications^20^, increased inflammatory response^21^, and aggravation of muscular weakness^22^. Patients of mechanical ventilation dependence often need ICU admission which will in turn further increase hospital stay and cost.

Surgical factors associated with pulmonary complications include extent and location of the surgical incision^23–25^. In our previous study, we demonstrated that patients undergoing open surgery for transverse colon cancer had higher risk of ICU admission, mechanical ventilation dependence, and hospitalization for pneumonia as compared to patients undergoing laparoscopic surgery^14^. The difference was attributed to the longer incision placed in the upper abdomen in the open surgery. In surgery for left-sided, especially the sigmoid colon cancer, the incisions were placed over the middle and lower abdomen for both open and laparoscopic procedures. The two groups of patients were also comparable with regard to the rates of underlying chronic pulmonary disease. Therefore the difference might be explained mainly in the size of the wounds. The consequence of larger wounds was also evident from the increased opioid usage in the group of open surgery. Both pain and the subsequent increased use of opioid may increase the risk of pulmonary complications^26^.

In our study, laparoscopic surgery for left-sided colon cancer was associated with higher likelihood of prolonged operation time, which was compatible with most of previous reports^3,7,11^. Although prolonged operation was suggested to be a potential risk factor for development of postoperative pulmonary complications, our result that laparoscopic surgery was associated with lower risk of ICU admission and mechanical ventilation suggested that the contribution of prolonged operation time was out-weighed by other factors such as incision size and pain^18,25,26^.

Although parameters of oncological outcomes such as tumor staging and timing of recurrence were not available in the NHIRD, the comparable overall survival of the patients undergoing open and laparoscopic procedures implied that the equivalent survival outcomes observed in previous randomized trials could be achieved in the real world^4,7,8,10,11,16^. Furthermore, we performed propensity score matching to reduce the bias associated with the observational nature of the study. The results attained were comparable with those from the original unmatched groups. Although increased cost is a major drawback of laparoscopic surgery, the decreased adverse events and higher possibility of shortened hospital stay in the laparoscopic group may also compensate for the potential increased cost from surgical instruments in this group of patients.

The main strength of this study is the use of a nationwide population-based dataset from Taiwan which launched a universal NHI program that covered more than 99% of its population. Therefore this study excluded the influence of insurance status which had been demonstrated to be an important confounding factor influencing the care of patients with colon cancer^27^. At the time of the sampling period, laparoscopic surgery for colon cancer has become a mature technique. Therefore, the effect of learning curve on outcomes could be obviated^28,29^. Nevertheless, the consumables used in the laparoscopic procedures were not fully covered at the time of data registration. In addition, hospitals of various statuses were not evenly distributed in Taiwan. As these factors would influence the patients’ preference and accessibility to the laparoscopic procedures, we included monthly income and residence area in the analysis of the data and found no difference between the two groups.

However, there are still some limitations remained in this study. Details in history of previous abdominal surgery, emergency status of the operation, cancer stage, postoperative complications, and body mass index were not available from this dataset. These factors might have impact on postoperative outcomes. For instance, open procedures might be performed more frequently in patients with an advanced or acute disease, biasing the comparison between groups. Moreover, parameters of short-term oncological outcomes such as tumor size, number of lymph node retrieved, and surgical margin are important to assess the oncological adequacy of a novel surgical procedure. These parameters were not accessible in the NHIRD. The dataset used in this study did not allow us to preclude the confounding effect of these factors. In addition, the dedicated code for laparoscopic descending and sigmoid colon cancer resection did not exist in the NHI program until the year 2007. Variation in declaration policy among hospitals and surgeons may result in under-reporting of laparoscopic procedures and subsequent selection bias.

By reviewing a nationwide population-based database, this study found that laparoscopic left-sided colon cancer resection was associated with improved perioperative outcomes and reduced risk of adverse events. The long-term survival was equivalent to that achieved by conventional open procedures. It is also important that adequate hospital quality and volume is required to realize the benefits of laparoscopic surgery.

## Supplementary information

Supplementary tables S1 and S2.

## Data Availability

The datasets during and/or analysed during the current study are available from the corresponding author on reasonable request.
