# Author Correction: Comparison of clinical outcomes between laparoscopic and open surgery for left-sided colon cancer: a nationwide population-based study

**DOI:** 10.1038/s41598-021-88762-y

**Published:** 2021-04-21

**Authors:** Yu-Min Huang, Yuan-Wen Lee, Yan-Jiun Huang, Po-Li Wei

**Affiliations:** 1 Department of Surgery, School of Medicine, College of Medicine, Taipei Medical University, Taipei, Taiwan; 2 Division of Gastrointestinal Surgery, Department of Surgery, Taipei Medical University Hospital, Taipei Medical University, Taipei, Taiwan; 3 Cancer Research Center, Taipei Medical University Hospital, Taipei Medical University, Taipei, Taiwan; 4 Department of Anesthesiology, Taipei Medical University Hospital, Taipei, Taiwan; 5 Department of Anesthesiology, School of Medicine, College of Medicine, Taipei Medical University, Taipei, Taiwan; 6 Division of Colorectal Surgery, Department of Surgery, Taipei Medical University Hospital, Taipei Medical University, Taipei, Taiwan; 7 Translational Laboratory, Department of Medical Research, Taipei Medical University Hospital, Taipei Medical University, Taipei, Taiwan; 8 Graduate Institute of Cancer Biology and Drug Discovery, Taipei Medical University, Taipei, Taiwan

Correction to: *Scientific Reports* 10.1038/s41598-019-57059-6, published online 09 January 2020

The original version of this Article contained an error in Affiliation 1, which was incorrectly given as ‘Department of Surgery, College of Medicine, Taipei Medical University, Taipei, Taiwan’. The correct affiliation is listed below:

Department of Surgery, School of Medicine, College of Medicine, Taipei Medical University, Taipei, Taiwan.

This error has now been corrected in the HTML and PDF versions of the Article, and in the accompanying Supplemental Material.

