# Supplementary material for: Comparison of clinical outcomes between laparoscopic and open surgery for left-sided colon cancer: a nationwide population-based study
Source: Sci Rep. 2020 Jan 9;10:75. doi: 10.1038/s41598-019-57059-6 (PMC6952445; doi:10.1038/s41598-019-57059-6)
Supplement: Supplementary file 1 [file 41598_2019_57059_MOESM1_ESM.docx]

**Comparison of clinical outcomes between laparoscopic and open surgery for left-sided colon cancer: a nationwide population-based study**

Yu-Min Huang, MD, PhD^1,2,3^

Yuan-Wen Lee, MD, PhD^4,5^

Yan-Jiun Huang, MD, PhD^1,6^

Po-Li Wei, MD, PhD^1,3,6,7,8,^

^1^Department of Surgery, School of Medicine, College of Medicine, Taipei Medical University, Taipei, Taiwan

^2^Division of Gastrointestinal Surgery, Department of Surgery, Taipei Medical University Hospital, Taipei Medical University, Taipei, Taiwan

^3^Cancer Research Center, Taipei Medical University Hospital, Taipei Medical University, Taipei, Taiwan

^4^Department of Anesthesiology, Taipei Medical University Hospital, Taipei, Taiwan

^5^Department of Anesthesiology, School of Medicine, College of Medicine, Taipei Medical University, Taipei, Taiwan

^6^Division of Colorectal Surgery, Department of Surgery, Taipei Medical University Hospital, Taipei Medical University, Taipei, Taiwan

^7^Translational Laboratory, Department of Medical Research, Taipei Medical University Hospital, Taipei Medical University, Taipei, Taiwan

^8^Graduate Institute of Cancer Biology and Drug Discovery, Taipei Medical University, Taipei, Taiwan

Table S1. Perioperative outcomes after propensity score matching

|  | Total (N = 414) |  | Open surgery (N = 276) |  | Laparoscopic surgery (N = 138) |  |  |
| --- | --- | --- | --- | --- | --- | --- | --- |
| Variables | N (%) or |  | N (%) or |  | N (%) or |  | *P* value |
| Length of hospital stay (Days) |  |  |  |  |  |  | < 0.0001 |
| ≤10 | 148 | (35.8) | 75 | (27.3) | 73 | (52.9) |  |
| >10 | 266 | (64.3) | 201 | (72.8) | 65 | (47.1) |  |
| Surgery time (Hours) |  |  |  |  |  |  | < 0.0001 |
| ≤4 | 242 | (58.5) | 185 | (67.0) | 57 | (41.3) |  |
| >4 | 172 | (41.6) | 91 | (33.0) | 81 | (58.7) |  |
| Opioid use (MEQ) |  |  |  |  |  |  | 0.004 |
| ≤15 | 232 | (56.0) | 141 | (51.1) | 91 | (65.9) |  |
| >15 | 182 | (44.0) | 135 | (48.9) | 47 | (34.1) |  |
| Blood transfusion |  |  |  |  |  |  | 0.02 |
| Yes | 101 | (24.4) | 77 | (27.9) | 24 | (17.4) |  |
| No | 313 | (75.6) | 199 | (72.1) | 114 | (82.6) |  |
| ICU admission |  |  |  |  |  |  | < 0.0001 |
| Yes | 96 | (23.2) | 80 | (29.0) | 16 | (11.6) |  |
| No | 318 | (76.8) | 196 | (71.0) | 122 | (88.4) |  |
| Mechanical ventilation |  |  |  |  |  |  | 0.0002 |
| Yes | 70 | (16.9) | 60 | (27.4) | 10 | (7.3) |  |
| No | 344 | (83.1) | 216 | (78.3) | 128 | (92.8) |  |

Abbreviation: MEQ, morphine equivalent dose.

Table S2. Relative risks of clinical outcomes after propensity score matching

|  | Blood transfusion | ICU admission | Mechanical ventilation |
| --- | --- | --- | --- |
| Variables | aOR (95% CI) | aOR (95% CI) | aOR (95% CI) |
| Open surgery | 1.92 (1.10-3.37) | 3.63 (1.94-6.81) | 3.81 (1.82-7.97) |
| Age, years |  |  |  |
| <55 | 1 | 1 | 1 |
| 55-64 | 1.06 (0.47-2.40) | 0.97 (0.40-2.37) | 1.22 (0.41-3.64) |
| 65-74 | 1.50 (0.68-3.29) | 2.39 (1.06-5.39) | 3.89 (1.46-10.34) |
| ≥75 | 4.39 (1.97-9.79) | 4.88 (2.10-11.35) | 5.02 (1.84-13.73) |
| Male | 0.53 (0.32-0.89) | 1.09 (0.63-1.89) | 0.80 (0.44-1.44) |
| Comorbidity |  |  |  |
| Congestive heart failure | 0.38 (0.05-2.75) | 0.32 (0.05-1.96) | 1.97 (0.36-10.74) |
| Cerebrovascular disease | 0.59 (0.22-1.55) | 1.07 (0.43-2.64) | 1.05 (0.40-2.73) |
| Chronic pulmonary disease | 1.26 (0.59-2.69) | 0.85 (0.38-1.88) | 1.23 (0.55-2.77) |
| Renal disease | 5.75 (1.92-17.19) | 1.78 (0.53-6.04) | 0.83 (0.20-3.40) |
| Liver disease | 0.86 (0.30-2.51) | 0.70 (0.23-2.18) | 1.28 (0.41-4.03) |
| Metastasis | 4.36 (1.29-14.70) | 2.35 (0.62-8.99) | 6.37 (1.76-23.08) |
| Hypertension | 0.85 (0.49-1.47) | 1.01 (0.58-1.76) | 1.06 (0.58-1.96) |
| Diabetes mellitus | 1.52 (0.79-2.92) | 0.93 (0.46-1.89) | 0.84 (0.39-1.81) |
| Hospital |  |  |  |
| Medical center | 1 | 1 | 1 |
| Metropolitan hospital | 1.87 (1.10-3.18) | 4.35 (2.47-7.66) | 1.91 (1.06-3.44) |
| Local community hospital | 7.55 (2.36-24.14) | 4.10 (1.23-13.63) | 1.15 (0.27-4.92) |

Abbreviation: aOR, adjusted odds ratio.
